# Prevalence and Factors Associated with Insomnia Among Healthcare Workers in Kazakhstan During the COVID-19 Pandemic: A Cross-Sectional Study

**DOI:** 10.3390/medicina62061094

**Published:** 2026-06-04

**Authors:** Nurila Aryntayeva, Kuanysh Shonbay, Tulay Koru-Sengul, Fatima Bagiyarova, Gulshara Aimbetova, Guoyan Zhang, Saltanat Umbetkulova, Abzal Zhumagaliuly, Venera Baisugurova, Indira Karibayeva

**Affiliations:** 1 Department of Clinical Disciplines, Al-Farabi Kazakh National University, Almaty 050040, Kazakhstan; aryntayeva.n@kaznmu.kz; 2 Department of Public Health, Asfendiyarov Kazakh National Medical University, Almaty 050012, Kazakhstan; aimbetova.g@kaznmu.kz; 3 Department of Public Health Sciences, Leonard M. Miller School of Medicine, University of Miami, Miami, FL 33136, USA; tsengul@med.miami.edu; 4 Higher School of Psychology, Turan University, Almaty 050013, Kazakhstan; 5 Department of Communication Skills, Asfendiyarov Kazakh National Medical University, Almaty 050012, Kazakhstan; bagiyarova.f@kaznmu.kz; 6 Florida Department of Health in Miami-Dade County, Miami, FL 33125, USA; guoyan.zhang@flhealth.gov; 7 School of Medicine, Nazarbayev University, Astana 010000, Kazakhstan; saltanat.umbetkulova@nu.edu.kz; 8 B. Atchabarov Scientific-Research Institute of Fundamental and Applied Medicine, Asfendiyarov Kazakh National Medical University, Almaty 050012, Kazakhstan; zhumagali.a@kaznmu.kz; 9 Department of Biostatistics and Foundations of Scientific Research, Asfendiyarov Kazakh National Medical University, Almaty 050012, Kazakhstan; baysugurova.v@kaznmu.kz; 10 Department of Research Management, JSC Research Institute of Cardiology and Internal Diseases, Almaty 050012, Kazakhstan; 11 Jiann-Ping Hsu College of Public Health, Georgia Southern University, Statesboro, GA 30460, USA

**Keywords:** COVID-19, healthcare worker, medical worker, insomnia, mental health

## Abstract

*Background and Objectives*: Healthcare workers (HCWs) involved in the COVID-19 response are at increased risk of mental health disturbances, including sleep disorders. This study aimed to assess the prevalence and predictors of insomnia among HCWs in Almaty, Kazakhstan. *Materials and Methods*: A cross-sectional study was conducted between 11 and 26 September 2021, including 553 HCWs. Insomnia symptoms were assessed using the Insomnia Severity Index (ISI). The primary binary outcome was defined as an ISI score ≥ 10, while ISI ≥ 15 was used descriptively to indicate moderate-to-severe insomnia symptoms. Associations were evaluated using chi-square tests and multivariable logistic regression models. Statistical significance was set at *p* < 0.05. *Results*: Overall, 38.10% of HCWs with complete ISI data had insomnia symptoms based on the predefined ISI ≥ 10 threshold. In multivariable analysis, Kazakh nationality (AOR = 2.11, 95% CI: 1.05–4.23), advanced education (AOR = 2.03, 95% CI: 1.13–3.65), physician role (AOR = 4.92, 95% CI: 1.25–19.30), and working with COVID-19 patients for >1 year (AOR = 2.28, 95% CI: 1.33–3.89) were significantly associated with increased odds of insomnia. *Conclusions*: Insomnia symptoms were common among surveyed HCWs during the COVID-19 pandemic and were associated with selected demographic and occupational characteristics, including professional role, education level, and duration of work with COVID-19 patients. These findings highlight the need for targeted mental health interventions and structural support systems for HCWs in Kazakhstan.

## 1. Introduction

The COVID-19 pandemic has profoundly impacted global health systems, causing unprecedented strain on healthcare resources and personnel. As of July 2024, the World Health Organization (WHO) reports over 700 million confirmed cases and approximately 7 million deaths globally due to COVID-19 [1]. Healthcare professionals (HCWs), who are at the frontline of the response, have faced significant physical and psychological challenges throughout the pandemic. These challenges have included increased workloads, prolonged exposure to high-stress environments, and heightened risk of infection, all of which have substantially affected their mental health and well-being [2].

One critical aspect of health that has been significantly impacted among HCWs is sleep quality. Insomnia, defined as difficulty falling asleep, staying asleep, or experiencing non-restorative sleep, has become increasingly prevalent among HCWs during the pandemic. Studies indicate that poor sleep quality not only affects cognitive function and emotional regulation but also has long-term implications for physical health, including increased risks of cardiovascular diseases and immune dysfunction [3,4]. The importance of sleep for maintaining the cognitive and physical performance of HCWs, who are essential for managing the pandemic, cannot be overstated [5].

Recent data highlights the severity of sleep disturbances among HCWs globally. A meta-analysis by Pappa et al. (2020) reported that the prevalence of insomnia among HCWs during the COVID-19 pandemic ranged from 23.6% to 37.9%, significantly higher than in the general population [6]. This increase in insomnia is attributed to various pandemic-related stressors, including the fear of contracting and spreading the virus, extended working hours, and the emotional toll of dealing with a high number of critically ill and dying patients [7].

In Kazakhstan, the healthcare system has faced considerable challenges during the COVID-19 pandemic. As of July 2024, Kazakhstan has reported over 1.5 million confirmed COVID-19 cases and approximately 20,000 deaths, according to Worldometer [8]. The surge in cases has overwhelmed healthcare facilities and placed immense pressure on HCWs, exacerbating existing issues such as inadequate staffing and limited resources [9]. Despite these challenges, there has been limited research focused on the mental health and sleep quality of HCWs in Kazakhstan, creating a critical gap in understanding the full impact of the pandemic on this essential workforce.

Several factors contribute to the high prevalence of insomnia among HCWs during the COVID-19 pandemic. Long working hours and shift work disrupt circadian rhythms, leading to sleep disturbances [10]. The constant use of personal protective equipment (PPE) and adherence to stringent infection control protocols can also contribute to physical discomfort and stress, further impairing sleep quality [11]. Additionally, the psychological burden of witnessing patient suffering, the fear of infection, and concerns about family and loved ones’ safety add to the emotional stress experienced by HCWs [12].

Addressing sleep disturbances in HCWs is crucial for ensuring their well-being and maintaining the resilience of healthcare systems. Poor sleep quality has been associated with decreased job performance, impaired decision-making, and an increased risk of medical errors, which can have serious implications for patient care and safety [13]. Moreover, chronic insomnia can lead to long-term health issues, further reducing the capacity of HCWs to effectively respond to ongoing and future public health emergencies [14].

Interventions aimed at improving sleep quality among HCWs are essential for mitigating the negative impact of the COVID-19 pandemic on this critical workforce. Strategies such as providing mental health support, promoting adequate rest breaks, and implementing stress management programs can help alleviate some of the factors contributing to sleep disturbances [15]. Public health policies that recognize and address the mental health needs of HCWs are also vital for building a resilient healthcare system capable of withstanding future public health crises [16].

Despite growing global evidence, data on sleep disturbances among HCWs in Central Asia remain limited. In particular, Kazakhstan represents an underexplored context with unique healthcare system challenges. Therefore, this study aimed to estimate the prevalence of insomnia and identify demographic and occupational factors associated with insomnia among HCWs during the COVID-19 pandemic.

## 2. Materials and Methods

### 2.1. Study Design

This cross-sectional study was conducted between 11 and 26 September 2021, using an online survey to collect quantitative data from healthcare workers. The inclusion criteria included any healthcare professional working in hospitals or healthcare facilities designated as treatment centers for COVID-19 patients. Relevant services at these facilities encompassed medical evaluations for at-risk individuals, intensive care units, inpatient units, and other medical services specifically designed for the care of COVID-19 patients. Direct work with COVID-19 patients was not treated as an eligibility criterion.

### 2.2. Study Population

For participant selection, the prevalence of insomnia among healthcare workers during the COVID-19 pandemic was estimated to be 0.43 [17]. Based on this prevalence, a required sample size of 334 was calculated with a margin of error (d) of 0.05, from a population of 3218 [18]. The study used a non-probability convenience sampling strategy, and data were collected using SurveyMonkey.com software (SurveyMonkey Inc., San Mateo, CA, USA). Therefore, the study sample should be interpreted as a respondent sample of HCWs who were reachable and willing to participate in an online survey, rather than as a probability-based representative sample of all HCWs in Kazakhstan. All participants were provided with detailed information about the study’s objectives, methods, and consent forms. The study was conducted in accordance with the Declaration of Helsinki. The study protocol received ethical approval from the Institutional Review Board of Kazakh National Medical University in Almaty, Kazakhstan (approval number: 8/114). Written informed consent was obtained from all participants prior to participation.

### 2.3. Study Instruments

The study employed a structured questionnaire to gather information on demographic characteristics, work details, and perceived support from administration and colleagues. Current/recent insomnia symptoms were assessed using the Insomnia Severity Index (ISI), a seven-item instrument that evaluates the perceived severity and impact of insomnia symptoms. The ISI asks participants to rate the severity of difficulties with sleep onset, sleep maintenance, and early morning awakening; satisfaction with the current sleep pattern; the extent to which sleep problems interfere with daytime functioning; how noticeable the sleep problem is to others; and the level of distress or worry caused by the sleep problem. Each item is scored from 0 to 4, yielding a total score ranging from 0 to 28, with higher scores indicating more severe insomnia symptoms. For the primary analysis, insomnia symptoms were defined a priori as an ISI score ≥ 10, consistent with the threshold used to identify probable insomnia cases [19]. The internal consistency of the ISI in the present sample was high, with a Cronbach’s alpha of 0.87.

### 2.4. Statistical Data Analysis Methods

Data analysis was performed using SAS version 9.4 (SAS Institute Inc., Cary, NC, USA). Descriptive statistics were used to summarize the characteristics of healthcare workers and their mental health outcomes. The primary dependent variable was binary insomnia status, defined as ISI < 10 versus ISI ≥ 10. Participants with missing ISI scores were excluded from analyses involving the insomnia outcome. Univariable and multivariable logistic regression analyses were conducted to estimate odds ratios (ORs), adjusted odds ratios (AORs), and 95% confidence intervals (CIs). All key assumptions for logistic regression were assessed before model fitting. A two-sided *p*-value < 0.05 was considered statistically significant. Variables included in the regression models were selected based on evidence from the prior literature.

## 3. Results

The demographic characteristics of the HCWs in this study are detailed in Table 1. The respondent sample included 553 HCWs, with an average age of 35.88 ± 9.71 years. Accordingly, 83.5% of HCW’s were females and 88.43% identified themselves as Kazakhs. Most of the participants had attained at least a bachelor’s degree (69.08%) and were married (73.96%). In terms of professional experience, nearly half (49.01%) had been in practice for 5 to 10 years, and a significant portion were physicians (74.50%). Most respondents were based in Almaty (63.47%), while 36.53% reported working in other locations; however, the available location variable did not allow more detailed rural–urban or region-specific comparisons. At the time of the survey, 44.67% had been treating COVID-19 patients for more than a year. While 65.64% of HCWs reported no significant issues in managing COVID-19 patients, 34.36% experienced various challenges.

The sleep quality outcomes of HCWs are summarized in Table 2. Insomnia was present in 38.10% of participants, with a cut-off score of ≥10.

Table 3 presents the demographic characteristics of HCW’s contributing to insomnia through multiple logistic regression analysis. The multinomial regression analysis identified several significant factors associated with insomnia among healthcare workers during the COVID-19 pandemic. Kazakh nationality is significantly associated with higher odds of experiencing insomnia compared to other nationalities (AOR: 2.109, 95% CI: 1.050–4.234, *p* = 0.0360). Healthcare workers with an advanced graduate degree have significantly higher odds of experiencing insomnia compared to those with only a college degree (AOR: 2.028, 95% CI: 1.125–3.654, *p* = 0.0186). Physicians, compared to those in administrative/healthcare roles, are significantly more likely to experience insomnia (AOR: 4.919, 95% CI: 1.253–19.303, *p* = 0.0224). Healthcare workers with more than one year of experience working with COVID-19 patients have significantly higher odds of experiencing insomnia compared to those who did not work with COVID-19 patients (AOR: 2.277, 95% CI: 1.334–3.887, *p* = 0.0025). Those with one year or less of experience also show higher odds compared to those who did not work with COVID-19 patients (AOR: 1.883, 95% CI: 1.045–3.394, *p* = 0.0353).

## 4. Discussion

His study found a substantial burden of insomnia symptoms among HCWs who participated in the online survey (38.10%). These results underscore the considerable psychological challenges experienced by HCWs, highlighting the critical need for tailored mental health support interventions. Several mechanisms reported in the prior literature may help contextualize the observed burden of insomnia among HCWs during the COVID-19 pandemic, including extended working hours, shift work, PPE-related discomfort, fear of infection, and psychological stress [20,21]. However, these factors were not directly measured in the present study and should therefore be interpreted as plausible contextual explanations rather than empirically tested predictors. Additionally, the psychological burden of treating severely ill patients, fear of infection, and concerns for family safety exacerbate emotional stress among HCWs [22,23]. The observed association with physician role may reflect higher clinical responsibility and prolonged exposure to critically ill patients. However, this finding should be interpreted cautiously because physicians constituted most of the sample, whereas administrative and other healthcare roles were represented by small numbers, likely contributing to the wide confidence interval and possible instability of the estimate.

Kazakhstan experienced a significant surge in COVID-19 cases in July 2021, with the highest number of daily confirmed cases reaching a seven-day average of 14,234, as reported by the Johns Hopkins Coronavirus Resource Center [24]. This dramatic increase in cases subsequently led to a rise in the seven-day average of daily deaths, peaking at 259 by mid-August. This surge placed an extraordinary burden on HCWs, exacerbating the already challenging conditions they faced [24]. Interestingly, previous studies observed that anxiety levels among HCWs in Kazakhstan were lower than the global average, while the prevalence of sleep disorders was comparable to the worldwide rates [25]. The notable resilience displayed by HCWs can be partly attributed to substantial social support during the pandemic [26]. Government interventions, including financial aid, additional social benefits such as increased availability of kindergarten places, and public recognition initiatives like street art and social acknowledgment events, likely played a crucial role in fostering this resilience [26].

Kazakh nationality was associated with insomnia symptoms in the adjusted model. This finding should be interpreted cautiously and should not be understood as evidence of a biological or causal effect of ethnicity. Rather, nationality may be acting as a proxy for unmeasured social, cultural, occupational, or healthcare-system factors, such as differences in workplace setting, language environment, regional background, social support, household responsibilities, or patterns of perceived and reported distress [27,28,29,30]. Because these factors were not directly measured in the present study, the observed association should be considered exploratory and hypothesis-generating. Future studies should include more detailed measures of socioeconomic position, work setting, regional residence, language, social support, and cultural factors to better understand whether and why such differences occur.

The prevalence of insomnia observed in the present study is consistent with international evidence showing a substantial sleep burden among healthcare workers during the COVID-19 pandemic. Studies from China reported high levels of insomnia among frontline medical staff, particularly among nurses and professionals exposed to prolonged occupational stress, fear of infection, and high clinical workload [31]. Similar patterns were reported across European healthcare settings, where insomnia was frequently linked with burnout, psychological distress, and sustained pandemic-related work pressure [32]. Evidence from low- and middle-income countries also indicates that sleep disturbances among healthcare workers were amplified by resource constraints, workforce shortages, and limited access to structured mental health support [33]. Taken together, these findings suggest that the insomnia burden observed among surveyed healthcare workers in Kazakhstan may be related to both individual characteristics and broader occupational and health-system stressors common to pandemic responses across diverse settings.

The sampling strategy may have influenced the observed prevalence in either direction. HCWs experiencing sleep problems may have been more motivated to participate in a survey on insomnia, which could lead to overestimation. Conversely, HCWs with the highest clinical workloads, limited time, or reduced access to online survey platforms may have been less likely to respond, potentially leading to underestimation. Therefore, the prevalence estimate should be interpreted cautiously, while the associations observed in the regression analysis should be considered exploratory and hypothesis-generating.

The implications of poor sleep quality on mental health are significant. Insomnia can aggravate existing conditions of anxiety and depression, which have been prevalent among HCWs during the pandemic [34]. Prior studies among HCWs in Kazakhstan during the COVID-19 pandemic have shown that insomnia frequently co-occurs with symptoms of anxiety, depression, burnout, and psychological distress [35,36,37]. Therefore, the high burden of insomnia observed in the present study may indicate a need for broader occupational health attention to sleep and psychological well-being. Chronic sleep deprivation negatively impacts cognitive functions and decision-making abilities and increases the risk of medical errors, posing serious consequences for patient care and safety [38]. Furthermore, long-term insomnia is associated with various health issues, including cardiovascular diseases and weakened immune function [39]. Although the majority of the study participants were young and female, neither age nor gender emerged as a significant predictor of mental health issues among HCWs in this study. The significant predictors were predominantly occupational factors, including work experience, duration of working with COVID-19 patients, possession of advanced post-college degrees, and specific job roles. Ethnicity was the only non-occupational factor found to be significant.

Addressing sleep disturbances in HCWs is essential. Implementing mental health support, promoting adequate rest breaks, and introducing stress management programs can alleviate some of the factors contributing to sleep disturbances [40]. Organizational interventions such as flexible scheduling and providing access to mental health resources are crucial for supporting HCWs’ well-being [41]. Future occupational health programs for HCWs should consider integrating sleep-health assessment with broader psychological support, although further studies with direct mental health measures are needed to identify the specific psychological needs associated with insomnia [42]. Kazakhstan, as of 2020, has established a standalone mental health policy and plan but lacks comprehensive mental health legislation [43]. Additionally, the country does not have specific policies or plans targeting suicide prevention or work-related mental health prevention and promotion programs. This includes the absence of mental health and psychological components in emergency preparedness and risk reduction strategies [43].

This study has several limitations that must be acknowledged. First, its cross-sectional design restricts our ability to draw causal relationships between demographic factors and mental health outcomes. Additionally, the use of convenience sampling could introduce selection bias, potentially limiting the generalizability of the results to all HCWs in Kazakhstan. The reliance on self-reported questionnaires may lead to response biases, affecting the accuracy of the reported mental health data. Moreover, the duration of insomnia symptoms was not assessed; therefore, we could not distinguish transient sleep disturbances from persistent or chronic insomnia. Finally, the multivariable regression model may have been affected by sparse data in some categories, particularly occupation, and by the number of predictors relative to the number of insomnia cases. Although predictors were selected a priori and multicollinearity diagnostics were performed, some estimates had wide confidence intervals and should be interpreted cautiously. Future studies with more balanced samples and longitudinal data are needed to confirm these associations.

## 5. Conclusions

This study demonstrates a substantial burden of insomnia among surveyed healthcare workers during the COVID-19 pandemic, with 38.10% of participants reporting clinically relevant symptoms. The findings indicate that insomnia symptoms were statistically associated with selected occupational and demographic characteristics, including professional role, level of education, and duration of work with COVID-19 patients. Because of the cross-sectional design, these findings should be interpreted as associations rather than causal effects. Future studies should include direct measures of workload, shift work, PPE burden, perceived infection risk, and workplace support to clarify potentially modifiable mechanisms. These results underscore the substantial psychological and physiological burden placed on healthcare workers during large-scale public health emergencies. From a public health perspective, the high burden of insomnia has important implications for both workforce sustainability and patient safety. Sleep disturbances are known to impair cognitive performance, decision-making, and clinical efficiency, thereby increasing the risk of medical errors and reducing the overall effectiveness of healthcare systems under stress. In this context, addressing sleep health should be considered a critical component of healthcare system resilience. Targeted interventions are urgently needed, including organizational strategies such as workload optimization, structured shift scheduling, and guaranteed rest periods, as well as individual-level support through mental health services and stress management programs. At the policy level, integrating mental health and sleep support into emergency preparedness frameworks may enhance the capacity of healthcare systems to respond effectively to future crises.

## Figures and Tables

**Table 1:** Demographic characteristics of patients.

Characteristics	Frequency (%) ^1^ or Mean ± SD
Age	35.88 ± 9.71
Gender	
Female	462 (83.54)
Male	89 (16.09)
Nationality	
Kazakhs	489 (88.43)
Others	64 (11.57)
Education	
Undergraduate or college degree	382 (69.08)
Graduate degree	171 (30.92)
Marital Status	
Not married	88 (15.91)
Married	409 (73.96)
Divorced/Widowed	53 (9.58)
Work experience (years)	
<5 years	109 (19.71)
5–10 years	271 (49.01)
>10 years	173 (31.28)
Occupation	
Administrative/Healthcare	29 (5.24)
Physician	412 (74.50)
Nurse	112 (20.25)
City of residence	
Other	202 (36.53)
Almaty	351 (63.47)
Duration of work with COVID-19 patients	
≤1 year	146 (26.40)
>1 year	247 (44.67)
No experience	160 (28.93)
Presence of challenges when working with COVID-19 patients	
None	363 (65.64)
Some challenges present	190 (34.36)

^1^—Percentages may not sum to 100% due to missing values.

**Table 2:** Sleep Quality Outcomes.

Outcome	Frequency (%) ^1^
Insomnia status based on the predefined ISI ≥ 10 threshold	
ISI < 10: No insomnia symptoms	273 (61.90)
ISI ≥ 10: Insomnia symptoms	168 (38.10)

^1^—Missing data were excluded from the regression analysis.

**Table 3:** Univariable and multivariable logistic regression models of factors associated with insomnia symptoms.

Insomnia (Ref. Category: Absent)							
Variable	Categories	Univariate Models			Multivariate Model		
		Odds Ratio	95% CI	*p*-Value	Adjusted Odds Ratio	95% CI	*p*-Value
Gender (Ref. category: male)	Female	1.096	0.643–1.869	0.736	1.144	0.637–2.054	0.653
Age Groups (Ref. category: 18–34)	35–39	1.540	0.751–3.158	0.239	1.364	0.523–3.561	0.526
	40–44	1.500	0.636–3.541	0.354	1.283	0.349–4.721	0.708
	45–54	1.707	0.906–3.214	0.098	1.301	0.400–4.229	0.662
	55+	2.048	1.061–3.953	0.033	1.543	0.461–5.165	0.482
Nationality (Ref. category: other)	Kazakh	1.724	0.902–3.295	0.099	2.109	1.050–4.234	**0.036**
Education (Ref. category: Undergraduate or college degree)	Graduate degree	1.296	0.855–1.964	0.222	2.028	1.125–3.654	**0.019**
Marital Status (Ref. category: single)	Divorced/Widowed	1.514	0.728–3.149	0.267	1.005	0.439–2.303	0.991
	Married	0.674	0.402–1.131	0.135	0.612	0.343–1.089	0.095
Experience (Ref. category: <5 years)	5–10 years	1.043	0.615–1.768	0.876	1.046	0.581–1.881	0.882
	>10 years	1.760	1.010–3.066	0.046	1.563	0.525–4.653	0.423
Specialty (Ref. category: Administrative/Healthcare)	Physician	2.428	0.789–7.471	0.122	4.919	1.253–19.303	**0.022**
	Nurse	2.206	0.670–7.262	0.193	1.929	0.469–7.940	0.363
Place of Work (Ref. category: other)	Almaty	1.020	0.685–1.518	0.923	0.893	0.579–1.378	0.611
Work Experience (Ref. category: Did not work)	≤1 year	1.902	1.096–3.300	0.022	1.883	1.045–3.394	**0.035**
	>1 year	2.407	1.470–3.940	0.0005	2.277	1.334–3.887	**0.003**
Problem with COVID (Ref. category: none)	Some challenges	1.452	0.976–2.161	0.065	1.130	0.728–1.753	0.586

## Data Availability

Data are available from the corresponding author, Indira Karibayeva (ik01379@georgiasouthern.edu), upon reasonable request.
